# The Preparation of Black Goji Berry Enzyme and Its Therapeutic Effect on Alcoholic Liver Injury in Mice

**DOI:** 10.3390/foods14030523

**Published:** 2025-02-06

**Authors:** Keshan Wang, Zhishan Zhang, Wenge Xu, Shuyuan Yang, Jing Zhao, Zeyu Wu, Wencheng Zhang

**Affiliations:** 1 School of Food and Biological Engineering, Hefei University of Technology, Hefei 203009, China; 2022171520@mail.hfut.edu.cn (K.W.); 2023171407@mail.hfut.edu.cn (W.X.); 2023111272@mail.hfut.edu.cn (S.Y.); zhaojing_0710@163.com (J.Z.); wuzeyu@hfut.edu.cn (Z.W.); 2 Department of Architectural Environmental Art, Xi’an Academy of Fine Arts, No. 100 South Section of Hanguang Road, Xi’an 710065, China; zhangzhishan_1012@163.com; 3 Engineering Research Center of Bio-Process of Ministry of Education, School of Food and Biological Engineering, Hefei University of Technology, No. 420 Feicui Road, Hefei 230001, China

**Keywords:** antioxidant capacity, ALD, black goji berries, high acyl gellan gum

## Abstract

This study aimed to prepare a black goji berry enzyme (BGBE) using high acyl gellan gum as a substitute for aqueous slurry, followed by fermentation with Saccharomyces cerevisiae (SC) for 48 h, pasteurization, and subsequent fermentation with Lactobacillus plantarum (SC) for 48 h to obtain the optimal BGBE sample. The anthocyanin content and in vitro antioxidant activity were significantly enhanced. The primary objective of this study was to evaluate the potential therapeutic effect of BGBE on alcoholic liver injury (ALD) in mice. An animal model of alcoholic liver injury was established, and the levels of alanine aminotransferase (ALT), aspartate aminotransferase (AST), triglycerides (TG), total cholesterol (TC), malondialdehyde (MDA), superoxide dismutase (SOD), alcohol dehydrogenase (ADH), and aldehyde dehydrogenase (ALDH) in the serum and liver were analyzed. Furthermore, histopathological examination was performed using hematoxylin–eosin staining. The results indicated that BGBE significantly improved the liver histopathological condition in mice, markedly reducing the serum levels of ALT, AST, TG, TC, and the hepatic MDA levels (*p* < 0.05), while significantly increasing the levels of SOD, ADH, and ALDH (*p* < 0.05). The therapeutic effect of BGBE on alcoholic liver injury appears to be associated with its antioxidant properties.

## 1. Introduction

Excessive alcohol consumption is a major global health issue [1], with alcoholic liver injury (ALD) being a leading cause of liver failure, significantly increasing morbidity and mortality rates worldwide [2]. When ethanol enters the body, 80–90% of it is metabolized by alcohol dehydrogenase into acetaldehyde, which is then converted into acetate by acetaldehyde dehydrogenase [3]. Acetate combines with acetyl-CoA and enters the citric acid cycle, eventually becoming carbon dioxide and water for excretion [4]. However, chronic or excessive alcohol consumption raises ethanol levels in the body [5], while ADH activity remains unchanged. Excessive ethanol inhibits ADH, leading to ethanol accumulation in the liver and the development of ALD. Additionally, ethanol metabolism reduces NAD^+^ to reducing NADH, thereby increasing the NADH/NAD^+^ ratio [6]. This results in the accumulation of electrons and leakage at complexes I and III of the mitochondrial respiratory chain, which in turn promotes the generation of reactive oxygen species (ROS) [7]. The reduction in intracellular antioxidants causes oxidative stress, resulting in further liver damage [8]. Furthermore, genetic polymorphisms in ALDH and low enzyme activity can elevate acetaldehyde levels. Acetaldehyde, a highly toxic metabolite, is far more harmful than ethanol [9]. It damages mitochondria and microtubules in liver cells [10], generates ROS, and worsens oxidative stress-induced damage [11]. Currently, chemical drugs are the main treatment for ALD, but their toxic side effects limit long-term use [12]. Consequently, plant-based enzymes derived from the fermentation of fruits, vegetables, grains, and herbs by lactic acid bacteria, yeast, and other microorganisms have emerged as a promising alternative. These enzymes enhance antioxidant activity, support liver detoxification, and are safe for long-term use.

In recent years, the functionality of probiotics has attracted widespread attention, leading to the development of various probiotic enzymes aimed at improving human health. Zhao et al. used lactic acid bacteria to ferment a jujube and goji berry mixture, significantly enhancing its antioxidant capacity [13]. Fan et al. used Lactobacillus KP-3-fermented ginseng to treat alcohol-induced damage in C57 BL/6 N mice. They observed changes in liver fat degeneration, inflammation, and other injury indicators in different groups of mice. The results indicated that fermented ginseng could alleviate alcoholic liver injury and intestinal disorders by modulating the gut microbiota [14]. Probiotics decompose various compounds in substrates such as fruits and herbs, producing bioactive substances such as organic acids, short-chain fatty acids, and phenolic compounds. Some probiotics are capable of generating enzymes like superoxide dismutase (SOD) and lipase, and the activity of these enzymes is enhanced by their metabolic products. Additionally, the fermentation process can reduce sugar content and decrease antinutritional factors such as alkaloids, tannins, and oxalates [15].

The black goji berry (*Lycium ruthenicum* Murr., LRM) is widely recognized in China as a high-quality resource with both medicinal and dietary value [16]. It is rich in bioactive compounds such as polysaccharides, carotenoids, anthocyanins, and polyphenols [17], and has significant antioxidant and liver-protective effects [18]. Notably, black goji berry is hailed as the “king of anthocyanins” [19], with its anthocyanin content being 10.7, 4.9, 4.5, 1.9, and 1.8 times higher than that of grapes, raspberries, purple cabbage, purple sweet potatoes, and blueberries, making it an extremely potent source of antioxidants [20]. However, anthocyanins are highly unstable under natural conditions and prone to degradation. Xu et al. found that high acyl gellan gum could provide better beverage stability through stricter molecular association, significantly inhibiting the fading of anthocyanin pigments [21]. Liu et al. demonstrated that in a gelatin/gellan gum composite system, the ratio of gelatin to gellan gum of 8:2 (H2) exhibited the most compact and stable structure [22]. Therefore, adding high acyl gellan gum (HA) produced through the fermentation of *Sphingomonas* can bind with anthocyanins, forming gellan gum/anthocyanin composite films [23], thereby improving the stability of anthocyanins via acylation, as acylated anthocyanins are more stable than non-acylated ones. In addition, black goji berry contains a large amount of polyphenolic compounds, which exhibit excellent antioxidant activity within the plant.

In this study, *Saccharomyces cerevisiae* (SC) and *Lactobacillus plantarum* (LP) were used to sequentially ferment black goji berry. An animal model of alcoholic liver injury was established, and the therapeutic effects of the black goji berry enzyme on alcoholic liver injury were investigated through liver histopathological analysis, serum analysis, oxidative markers, and the measurement of alcohol dehydrogenase (ADH) and aldehyde dehydrogenase (ALDH) levels.

## 2. Materials and Methods

### 2.1. Materials

The black goji berry was provided by Qinghai Dehao Breeding and Cultivation Co., Ltd., (Qinghai, China). Saccharomyces cerevisiae (31,735) and Lactobacillus plantarum (22696) were purchased from the China Industrial Microorganism Strain Preservation and Management Center (Beijing, China). ADH and ALDH kits were purchased from Beijing Solarbio Science and Technology Co., Ltd., (Shanghai, China). SOD and malondialdehyde (MDA) kits were obtained from Wuhan Servicebio Technology Co., Ltd., (Wuhan, China). The alanine aminotransferase (ALT), aspartate aminotransferase (AST), triglycerides (TG), and total cholesterol (TC) kits were purchased from Ratro Life and Analytical Sciences Co., Ltd., (Shenzhen, China). LT100P high acyl gellan gum was purchased from Guangzhou Shengtong Trading Co., Ltd., (Guangzhou, China).

### 2.2. Methods

#### 2.2.1. Comparing Black Goji Berry Enzyme (FLRM) Samples Prepared by Different Fermentation Methods

Take an appropriate amount of clean, non-decayed black goji berries and mix them with a 0.14% high acyl gellan gum solution (HA) at a 1:4 ratio. The mixture is then blended, and the pH is adjusted to 4.3 using citric acid. Add a cellulase pectinase mixture (Shanghai Yuanye Biotechnology Co., Ltd., Shanghai, China) in a ratio of 6.89:3.11, with the total enzyme concentration being 0.1502%. The mixture is reacted at 30 °C for 2.45 h to obtain the slurry. Four different fermentation methods are designed as follows:

LP was inoculated and fermented for 12 h, 24 h, 36 h, 48 h, 60 h, 72 h, and 84 h, followed by inoculation of SC and fermentation for 84 h, 72 h, 60 h, 48 h, 36 h, 24 h, and 12 h, respectively (LP-SC).

LP was inoculated and fermented for 12 h, 24 h, 36 h, 48 h, 60 h, 72 h, and 84 h, then pasteurized (PT) before SC inoculation and fermented for 84 h, 72 h, 60 h, 48 h, 36 h, 24 h, and 12 h, respectively (LP-PT-SC).

SC was inoculated and fermented for 12 h, 24 h, 36 h, 48 h, 60 h, 72 h, and 84 h, followed by inoculation of LP and fermentation for 84 h, 72 h, 60 h, 48 h, 36 h, 24 h, and 12 h, respectively (SC-LP).

SC was inoculated and fermented for 12 h, 24 h, 36 h, 48 h, 60 h, 72 h, and 84 h, then pasteurized before LP inoculation and fermented for 84 h, 72 h, 60 h, 48 h, 36 h, 24 h, and 12 h, respectively (SC-PT-LP).

The activation rates of ADH and ALDH under different fermentation methods were measured using a multifunctional microplate reader (Synergy H1, BioTek, Winooski, VT, USA) according to the procedures provided in the ADH and ALDH assay kits.

(1) $$activation\;rate(\%)=\frac{A_{1}-A_{0}}{A_{0}},$$

A_0_: enzyme activity in the control group; A_1_: enzyme activity in the fermentation group.

#### 2.2.2. Measurement of Anthocyanin Content and In Vitro Antioxidant Activity

The anthocyanin content was analyzed using a modified pH differential method [24]. To 0.2 mL of the sample, pH 1.0 buffer (potassium chloride, 25 mmol/L) and pH 4.5 buffer (sodium acetate anhydrous, 400 mmol/L) were added separately. The mixture was allowed to react in the dark at room temperature for 50 min, and the absorbance was recorded at 535 and 700 nm using a UV-1800PC UV-Vis spectrophotometer (Hunan Xiangyi Laboratory Instrument Development Co., Ltd., Hunan, China). The anthocyanin concentration was reported as milligrams of petunidin-3-glucoside (P3) per gram and calculated as follows:

(2) $$X=\frac{A\times MW\times DF\times V}{\in \times \;1\times m\times 10},$$

X: The anthocyanin content in black goji berries, expressed as petunidin-3-glucoside equivalents (mg/100 g); A: the absorbance difference between the pH 1.0 and pH 4.5 buffers, calculated as A = (A535 − A700) pH1.0-(A535-A700)pH4.5; MW: 912.7, the average molecular weight of petunidin-3-glucoside (g/mol); DF: dilution factor; V: total volume of the extract (mL); ε: 29,591, the average molar extinction coefficient of petunidin-3-glucoside (L/(mol × cm)); 1: path length of the cuvette (1 cm); m: mass of the sample (g); 10: conversion factor to convert from g/kg to g/100 g.

The DPPH radical scavenging activity was measured based on a previously described method with modifications [25]. A 0.1 mmol/L DPPH–ethanol solution was prepared by dissolving DPPH in ethanol. Then, 2 mL of the DPPH solution was mixed with 1 mL of the sample and allowed to react in the dark for 30 min. The absorbance (A_t_) was measured at 517 nm using a multifunctional microplate reader. The absorbance of a blank (A_r_), where the DPPH solution was replaced with anhydrous ethanol, and a control (A_0_), where the sample was replaced with water, were also measured using the same procedure.

The ABTS radical scavenging activity was assessed based on a previously described method with modifications [26]. First, a 14 mmol/L potassium persulfate solution and a 7 mmol/L ABTS solution were prepared. Then, 88 µL of the potassium persulfate solution was mixed with 5 mL of the ABTS solution and allowed to react for 12 h to form an ABTS radical stock solution. The stock solution was diluted with anhydrous ethanol to prepare an ABTS working solution, which had an absorbance value between 0.68 and 0.72 at 734 nm. Next, 3 mL of the ABTS working solution was mixed with 1 mL of the sample and allowed to react in the dark for 6 min. The absorbance (A_t_) was measured at 734 nm using a multifunctional microplate reader. The absorbance of a blank (A_r_), where the ABTS working solution was replaced with anhydrous ethanol, and a control (A_0_), where the sample was replaced with water, were also measured using the same procedure.

The hydroxyl radical is among the most potent oxidants in aqueous solutions, and the detection method was adjusted accordingly [14]. An 8 mmol/mL ferrous sulfate solution, an 8 mmol/mL salicylic acid solution, and an 8.8 mmol/mL hydrogen peroxide solution were prepared. Subsequently, 1 mL each of ferrous sulfate solution, salicylic acid solution, hydrogen peroxide solution, and the sample were combined and incubated in the dark at 37 °C for 30 min. The absorbance (At) was recorded at 510 nm using a multifunctional microplate reader. The same procedure was followed to measure the absorbance of a blank (A_r_), where the hydrogen peroxide solution was replaced with distilled water, and a control (A_0_), where the sample was replaced with distilled water.

(3) $$Scavenging\;rate\left(\%\right)=100\times \left[1-\frac{A_{t}-A_{r}}{A_{0}}\right]$$

#### 2.2.3. Color Measurement

The color of the fermented black goji berry slurry was determined using a colorimeter (ZE7700, Nippon Denshoku Industries Co., Ltd., Tokyo, Japan) based on the three colors parameters: L* (lightness), a* (red/green), and b* (yellow/blue). The device was calibrated prior to use [27].

#### 2.2.4. Animal Treatment and Experimental Design

Sixty male C57BL/6 mice, approximately 5 weeks old and weighing 20–23 g, were purchased from Hefei Qingyuan Biotechnology Co., Ltd., Hefei, China. The animals were housed under controlled conditions with a temperature of 23 ± 0.5 °C, humidity at 55 ± 5%, and a 12 h light/dark cycle. They had ad libitum access to standard rodent chow and water. After a 7-day acclimatization period, the mice were randomly divided into six groups: blank control, model, silymarin positive control, low-dose LRM (L-LRM), medium-dose LRM (M-LRM), and high-dose LRM (H-LRM), with 10 mice in each group. The blank control group was given 6 mL/kg body weight (BW) of distilled water, while the other five groups received 6 mL/kg BW of 50% ethanol for 7 consecutive days. On the 7th day, at 9:00 AM, the blank control group was administered 6 mL/kg BW of distilled water, while the other five groups were given 6 mL/kg BW of 50% ethanol. Six hours later, the blank control and model groups were given 6 mL/kg BW of distilled water, the positive control group received 0.05 g/kg BW of silymarin, and the L-LRM, M-LRM, and H-LRM groups were administered 3.5 mL/kg, 7 mL/kg, and 10.5 mL/kg of fermented black goji berry, respectively. The treatment lasted for 21 days. Following the final administration, the mice were fasted for 12 h with free access to water. Subsequently, the mice were anesthetized with 5% chloral hydrate, blood was collected from the eyeball, and serum was separated by centrifugation at 6000 rpm for 5 min at 4 °C, then stored at −80 °C for future analysis. The mice were euthanized and dissected to obtain the liver, which was immediately washed with PBS (pH 7.4). A portion of the liver was stored in PBS at −80 °C for biochemical assays, while the other portion was fixed in 4% paraformaldehyde and stored at room temperature for histopathological examination. All animal experiments were conducted in compliance with the ethical guidelines of the Animal Ethics Committee of Hefei University of Technology.

#### 2.2.5. Determination of Blood Biochemical Parameters

The collected blood was centrifuged at 6000 rpm for 5 min at 4 °C to obtain the serum. The biochemical parameters, including ALT, AST, TG, and TC, were measured using a ChemRay-240 automatic biochemical analyzer (Ratro Life and Analytical Sciences Co., Ltd., Shenzhen, China), following the instructions provided in the corresponding reagent kits for each assay.

#### 2.2.6. Determination of ADH, ALDH, and Hepatic Oxidative Stress Parameters

Accurately weigh the animal tissue and, according to the weight (mg): volume (µL) ratio of 1:9, add 9 times the tissue weight volume of 0.9% saline. Homogenize the tissue under ice-cold conditions using a mechanical homogenizer to prepare a 10% tissue homogenate. Centrifuge at 2500–3000 rpm for 10 min, and collect the supernatant. The activities of ADH, ALDH, SOD, and malondialdehyde (MDA) are then measured according to the instructions provided in the respective reagent kits.

#### 2.2.7. Histopathological Examination

Liver tissues were fixed in 5% paraformaldehyde and then embedded in paraffin. The tissue sections were stained using a Hematoxylin and Eosin (H&E) staining kit (Servicebio Technology Co., Ltd., Wuhan, China). Pathological examination was performed using a Nikon ECLIPSE E100 upright optical microscope and Nikon DS-U3 imaging system (Nikon Corporation, Tokyo, Japan) to observe the tissue morphology and assess any pathological changes.

### 2.3. Statistical Analysis

Statistical analysis was performed using SPSS 27 software (Chicago, IL, USA). All data are presented as the mean ± standard deviation (SD). One-way analysis of variance (ANOVA) was used for inter-group comparisons. A *p*-value of <0.05 was considered statistically significant, and a *p*-value of <0.01 was considered highly statistically significant.

## 3. Results and Discussion

### 3.1. Optimal Fermentation Method

As shown in Table 1, different inoculation sequences and fermentation times, as well as various post-fermentation treatments, significantly increased the activation rates of ADH and ALDH (*p* < 0.05). The highest activation rates, 87.65% for ADH and 88.90% for ALDH, were observed in the SC 48 h-LTLT-LP 48 h sequence. LP was a strain that efficiently utilized a wide range of carbon sources. When SC and LP were co-fermented, SC’s growth and metabolism were inhibited because it could not access enough carbon sources [28]. When LP was added first, it produced a large number of acidic substances, creating an overly acidic environment that induced oxidative stress in yeast cells, altered intracellular MDA levels, and damaged cell membranes, leading to accelerated cell death [29]. In contrast, SC metabolism increased ethanol content in the fermentation broth, which inhibited the growth and metabolism of LP. However, this inhibitory effect was less significant than the impact of the acidic environment on SC [30]. Pasteurization after single-strain fermentation eliminates active cells, preventing interference with the secondary fermentation.

### 3.2. Changes in Color, Anthocyanins Content, and Antioxidant Activity of FLRM

Antioxidant performance was mainly demonstrated by the capacity to scavenge free radicals [31].

Table 2 shows the changes in DPPH, ABTS, and hydroxyl radical scavenging activities after fermentation with different solvents. Compared to the control group, all three radical scavenging activities increased significantly after fermentation (*p* < 0.05). In the LRM–water group, DPPH, ABTS, and hydroxyl radical scavenging activities increased by 22.01%, 23.2%, and 12.29%, respectively. In the LRM-HA group, the increases were 22.82%, 24.13%, and 18.35%, respectively. The LRM-HA group showed higher scavenging activity across all radicals compared to the LRM–water group, suggesting that the addition of HA enhances the antioxidant capacity of black goji berries [23].

Table 2 illustrates the changes in lightness (L*), redness (a*), and yellowness (b*) values before and after fermentation. A decrease in redness is generally considered an indication of anthocyanin loss during fermentation [21]. The a* value in the HA group was significantly higher than in the water group (*p* < 0.05). This corresponds to a reduction in the redness of the slurry, highlighting the protective effect of acyl groups against color fading during storage, demonstrating that HA significantly enhances anthocyanin stability.

### 3.3. Effects of Fermented Black Goji Berry on Serum Enzymes

Serum levels of ALT and AST are important indicators for assessing liver function. Both ALT and AST are primarily located in liver cells. When the liver is injured, the permeability of the cell membranes increases, causing the release of ALT and AST into the bloodstream, which results in elevated ALT and AST levels in the serum [32]. According to Table 3, the ALT and AST levels in the alcohol model group were significantly higher than those in the blank group (*p* < 0.01), indicating the successful establishment of the alcohol-induced liver injury model in mice. Compared to the alcohol model group, serum ALT and AST levels were significantly reduced in the positive control group (*p* < 0.01), L-LRM (*p* < 0.05), M-LRM (*p* < 0.01), and H-LRM (*p* < 0.01), with H-LRM being nearly identical to the positive control group. This suggests that fermented black goji berry has an inhibitory effect on ALT and AST levels.

Under normal conditions, the metabolism and synthesis of fatty acids in the liver are maintained in balance. Alcohol consumption affects the NAD⁺/NADH ratio in the body [33], which alters the composition of fatty acids, leading to metabolic disturbances and the accumulation of triglycerides (TG) and cholesterol (TC) [34]. According to Table 3, compared to the alcohol model group, the positive control group (*p* < 0.01), M-LRM (*p* < 0.01), and H-LRM (*p* < 0.01) showed significantly reduced serum TG levels. The TG content in the L-LRM group was also significantly reduced (*p* < 0.05), while there was no significant change in TC levels (*p* > 0.05). Moreover, the H-LRM group showed results nearly identical to the positive control group, suggesting that fermented black goji berry has an inhibitory effect on both TG and TC levels.

### 3.4. Effects of Fermented Black Goji Berry on Hepatic Oxidative Stress Parameters

Alcohol-induced oxidative stress is one of the key mechanisms underlying the development of ALD. After alcohol is absorbed into the bloodstream, it is primarily metabolized in the liver through three main pathways. The pathway involving ADH is the most significant, with approximately 90% of alcohol being converted into acetaldehyde and acetic acid by ADH and ALDH [35]. During this process, reactive oxygen species (ROS) are generated, leading to oxidative stress and a reduction in SOD levels. Additionally, this process accelerates the oxidation of polyunsaturated fatty acids, resulting in the formation of MDA [36]. According to Table 4, compared to the alcoholic model group, the positive control group (*p* < 0.01) and the high-dose LRM group (H-LRM, *p* < 0.05) showed significant increases in the activities of SOD and ALDH. The positive control group (*p* < 0.01), medium-dose LRM group (M-LRM, *p* < 0.01), and H-LRM group (*p* < 0.01) exhibited significantly increased ADH activity. In contrast, the positive control group (*p* < 0.01), M-LRM group (*p* < 0.05), and H-LRM group (*p* < 0.01) showed a significant decrease in MDA levels. These results suggest that fermented black goji berry attenuates the oxidative stress-induced damage caused by ethanol.

### 3.5. Hepatic Histopathological Analysis

**Figure 1:**
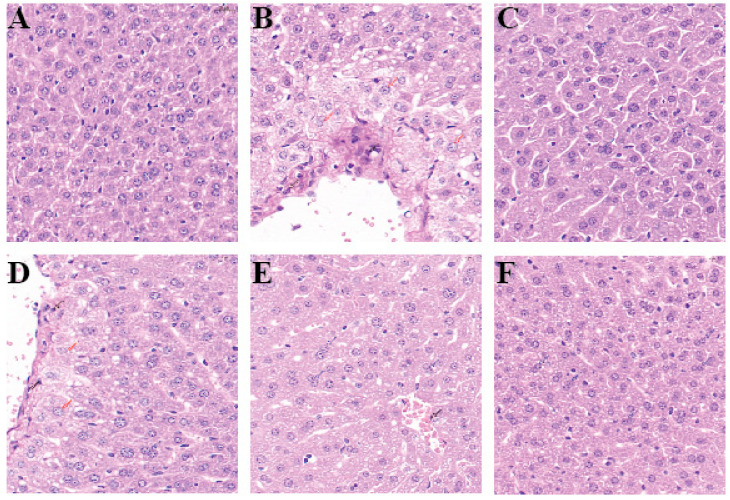
Groups: (**A**): normal control; (**B**): alcohol model control group; (**C**): positive control: Silymarin 0.05 g/kg; (**D**): L-LRM: 3.5 mL/kg; (**E**): M-LRM: 7 mL/kg; (**F**): H-LRM: 10.5 mL/kg. Red arrows indicate swollen liver cells; black arrows indicate inflammatory cell infiltration; blue arrows indicate binucleated liver cells. (BN). Scale bar: 20 μm.

The liver tissue of the normal control mice is intact, and the hepatocytes are arranged neatly (Figure 1A). In the model group, significant inflammatory cell infiltration, hepatocyte swelling, and fatty liver degeneration were observed (Figure 1B), indicating successful modeling of alcoholic liver injury. In the L-LRM and M-LRM groups, there was still fatty liver degeneration and a small amount of inflammatory cell infiltration, but binucleated hepatocytes began to appear, indicating that black goji berry enzyme played a therapeutic role (Figure 1D, E). For the H-LRM group, only mild fatty liver degeneration appeared, along with a higher number of binucleated hepatocytes (Figure 1F). This indicates that H-LRM: 10.5 mL/kg has the best therapeutic effect.

## 4. Conclusions

This study used high acylation gellan gum instead of aqueous solutions to reduce the natural loss of antioxidant compounds, particularly anthocyanins, and compared four fermentation methods to identify the optimal fermented black goji berry sample. The therapeutic effect of the sample on alcohol-induced liver injury in mice was also investigated. The results showed that high acylation gellan gum increased anthocyanin content by 1.01 mg/mL compared to the aqueous solution, demonstrating a protective effect. The optimal fermentation method was identified as SC 48h-PT-LP 48 h, under which the in vitro antioxidant activity was enhanced. Comprehensive analysis through serum ALT, AST, TG, TC levels, hepatic SOD, MDA, ADH, ALDH activities, and histopathological examination indicated that fermented black goji berry exhibited therapeutic effects on alcohol-induced liver injury in mice.

## Figures and Tables

**Table 1:** Changes in ADH and ALDH activation rates under different fermentation methods.

Sample	ADH Activation Rate (%)				ALDH Activation Rate (%)			
	LP-SC	LP-PT-SC	SC-LP	SC-PT-LP	LP-SC	LP-PT-SC	SC-LP	SC-PT-LP
12 h	62.85 ± 0.65 ^f^	78.12 ± 0.58 ^c^	65.19 ± 0.58 ^f^	56.28 ± 0.74 ^f^	62.25 ± 0.40 ^f^	75.96 ± 0.52 ^c^	61.21 ± 0.51 ^g^	56.82 ± 0.41 ^f^
24 h	75.21 ± 0.89 ^a^	83.12 ± 0.41 ^a^	68.80 ± 0.83 ^e^	65.68 ± 0.50 ^e^	76.74 ± 0.51 ^a^	80.53 ± 0.68 ^a^	65.95 ± 0.40 ^f^	63.79 ± 0.43 ^e^
36 h	69.95 ± 0.73 ^c^	73.23 ± 0.43 ^d^	72.08 ± 0.50 ^c^	75.51 ± 0.74 ^d^	71.53 ± 0.51 ^c^	71.28 ± 0.45 ^d^	71.82 ± 0.63 ^c^	75.23 ± 0.68 ^d^
48 h	71.77 ± 0.65 ^b^	79.73 ± 0.76 ^b^	78.25 ± 0.84 ^a^	88.52 ± 0.50 ^a^	74.65 ± 0.51 ^b^	77.71 ± 0.62 ^b^	79.35 ± 0.46 ^a^	88.52 ± 0.91 ^a^
60 h	63.93 ± 0.65 ^ef^	67.15 ± 0.73 ^f^	74.97 ± 0.66 ^b^	83.33 ± 0.74 ^b^	63.52 ± 0.56 ^e^	62.43 ± 0.51 ^g^	74.85 ± 0.54 ^b^	83.37 ± 0.51 ^b^
72 h	67.47 ± 0.40 ^d^	72.52 ± 0.67 ^d^	70.22 ± 0.50 ^d^	78.91 ± 0.74 ^c^	67.87 ± 0.30 ^d^	66.67 ± 0.51 ^e^	70.68 ± 0.80 ^d^	79.13 ± 0.80 ^c^
84 h	64.68 ± 0.90 ^e^	68.55 ± 0.65 ^e^	65.96 ± 0.74 ^f^	75.08 ± 0.82 ^d^	61.39 ± 0.57 ^g^	64.72 ± 0.51 ^f^	68.11 ± 0.63 ^e^	74.62 ± 0.46 ^d^

LP: *Lactobacillus plantarum*; SC: *Saccharomyces cerevisiae*; PT: pasteurized. Values with different letters in the same column indicate significant differences between groups by Fisher’s ANOVA (*p* < 0.05). Results are expressed as mean ± standard deviation (*n* = 3).

**Table 2:** Changes in anthocyanin content and antioxidant activity before and after fermentation under different solvents.

Sample	SC 48 h-PT-LP 48 h			
	NLRM–Water	FLRM–Water	NLRM-HA	FLRM-HA
Anthocyanin (mg/mL)	4.97 ± 0.08	6.33 ± 0.11	5.76 ± 0.09	7.34 ± 0.03
DPPH (%)	64.29 ± 0.57 ^d^	86.30 ± 0.49 ^b^	71.63 ± 0.41 ^c^	94.50 ± 0.16 ^a^
ABTS^+^ (%)	54.93 ± 0.83 ^d^	78.13 ± 0.46 ^b^	62.8 ± 0.40 ^c^	86.93 ± 0.46 ^a^
OH (%)	58.98 ± 0.29 ^d^	71.27 ± 0.54 ^b^	69.43 ± 0.53 ^c^	87.78 ± 0.36 ^a^
L*	27.00 ± 0.72 ^c^	24.95 ± 0.62 ^d^	30.28 ± 0.64 ^a^	28.62 ± 0.63 ^b^
a*	6.55 ± 0.38 ^c^	5.71 ± 0.21 ^d^	9.48 ± 0.29 ^a^	8.61 ± 0.22 ^b^
b*	−5.14 ± 0.35 ^b^	−3.51 ± 0.48 ^a^	−9.42 ± 0.99 ^d^	−7.87 ± 0.58 ^c^

Values with different letters in the same column indicate significant differences between groups by Fisher’s ANOVA (*p* < 0.05, *n* = 3).

**Table 3:** Effects of fermented black goji berry on serum biochemical parameters in alcohol-induced liver injury mice.

Group	ALT (U/L)	AST (U/L)	TG (mmol/L)	TC (mmol/L)
Normal control	40.91 ± 3.48	143.90 ± 2.85	0.82 ± 0.15	2.31 ± 0.20
Model	68.07 ± 4.45 ^b^	261.76 ± 8.06 ^b^	1.58 ± 0.04 ^b^	2.88 ± 0.11 ^b^
Positive control	46.75 ± 5.34 ^d^	165.59 ± 8.57 ^bd^	1.04 ± 0.08 ^bd^	2.44 ± 0.13 ^d^
L-LRM	61.08 ± 4.87 ^bc^	246.96 ± 6.13 ^bc^	1.45 ± 0.10 ^bc^	2.72 ± 0.12 ^b^
M-LRM	53.97 ± 4.38 ^bd^	220.68 ± 8.68 ^bd^	1.29 ± 0.05 ^bd^	2.61 ± 0.11 ^bd^
H-LRM	48.46 ± 6.10 ^ad^	183.51 ± 13.48 ^bd^	1.16 ± 0.08 ^bd^	2.52 ± 0.14 ^ad^

Compared to the normal control group: ^a^ *p* < 0.05; ^b^ *p* < 0.01; compared to the model group: ^c^ *p* < 0.05; ^d^ *p* < 0.01. Data are presented as mean ± standard deviation (*n* = 5). Groups: normal control; alcohol model control group; positive control: Silymarin 0.05 g/kg; L-LRM: 3.5 mL/kg; M-LRM: 7 mL/kg; H-LRM: 10.5 mL/kg.

**Table 4:** Effect of fermented black goji berry on hepatic oxidative indices in mice with alcoholic liver injury.

Group	SOD	MDA	ADH	ALDH
Normal control	697.16 ± 19.22	1.52 ± 0.17	0.0135 ± 0.0012	19.89 ± 2.50
Model	626.35 ± 52.97 ^b^	2.17 ± 0.20 ^b^	0.0044 ± 0.0015 ^b^	14.46 ± 3.20 ^b^
Positive control	688.64 ± 25.11 ^d^	1.66 ± 0.11 ^d^	0.0125 ± 0.007 ^d^	18.70 ± 2.06 ^d^
L-LRM	640.30 ± 36.04 ^a^	1.96 ± 0.21 ^b^	0.0057 ± 0.0017 ^b^	15.86 ± 2.05 ^b^
M-LRM	657.06 ± 24.11	1.88 ± 0.13 ^bc^	0.0077 ± 0.0008 ^bd^	16.67 ± 0.96 ^a^
H-LRM	679.31 ± 16.72 ^c^	1.76 ± 0.17 ^ad^	0.104 ± 0.0014 ^bd^	17.55 ± 1.93 ^c^

Compared to the normal control group: ^a^ *p* < 0.05; ^b^ *p* < 0.01; compared to the model group: ^c^ *p* < 0.05; ^d^ *p* < 0.01. Data are presented as mean ± standard deviation (*n* = 5). Groups: normal control; alcohol model control group; positive control: Silymarin 0.05 g/kg; L-LRM: 3.5 mL/kg; M-LRM: 7 mL/kg; H-LRM: 10.5 mL/kg.

## Data Availability

The original contributions presented in this study are included in the article. Further inquiries can be directed to the corresponding author.
